# Risk-adjusted cesarean section rates for the assessment of physician performance in Taiwan: a population-based study

**DOI:** 10.1186/1471-2458-6-246

**Published:** 2006-10-09

**Authors:** Chao-Hsiun Tang, Han-I Wang, Chun-Sen Hsu, Hung-Wen Su, Mei-Ju Chen, Herng-Ching Lin

**Affiliations:** 1 School of Health Care Administration, Taipei Medical University, Taipei, Taiwan; 2 Department of Obstetrics and Gynecology, College of Medicine, Taipei Medical University, Taipei, Taiwan; 3 Department of Obstetrics and Gynecology, Taipei Medical University – Wan Fang Hospital, Taipei, Taiwan; 4 Health Promotion Division, Department of Health, Taipei City Government, Taipei, Taiwan

## Abstract

**Background:**

Over the past decade, about one-third of all births nationwide in Taiwan were delivered by cesarean section (CS). Previous studies in the US and Europe have documented the need for risk adjustment for fairer comparisons among providers. In this study, we set out to determine the impact that adjustment for patient-specific risk factors has on CS among different physicians in Taiwan.

**Methods:**

There were 172,511 live births which occurred in either hospitals or obstetrics/gynecology clinics between 1 January and 31 December 2003, and for whom birth certificate data could be linked with National Health Insurance (NHI) claims data, available as the sample for this study. Physicians were divided into four equivalent groups based upon the quartile distribution of their crude (actual) CS rates. Stepwise logistic regressions were conducted to develop a predictive model and to determine the expected (risk-adjusted) CS rate and 95% confidence interval (CI) for each physician. The actual rates were then compared with the expected CS rates to see the proportion of physicians whose actual rates were below, within, or above the predicted CI in each quartile.

**Results:**

The proportion of physicians whose CS rates were above the predicted CI increased as the quartile moved to the higher level. However, more than half of the physicians whose actual rates were higher than the predicted CI were not in the highest quartile. Conversely, there were some physicians (40 of 258 physicians) in the highest quartile who were actually providing obstetric care that was appropriate to the risk. When a stricter standard was applied to the assessment of physician performance by excluding physicians in quartile 4 for predicting CS rates, as many as 60% of physicians were found to have higher CS rates than the predicted CI, and indeed, the CS rates of no physicians in either quartile 3 or quartile 4 were below the predicted CI.

**Conclusion:**

Overall, our study found that the comparison of unadjusted CS rates might not provide a valid reflection of the quality of obstetric care delivered by physicians, and may ultimately lead to biased judgments by purchasers. Our study has also shown that when we changed the standard of quality assessment, the evaluation results also changed.

## Background

The rise in cesarean section (CS) delivery rates in Western nations and many developing countries has become an issue of major public health concern. Over the past decade, about one-third of all births nationwide in Taiwan were delivered by CS, with the proportion having fluctuated between 32.39% and 34.47% [1]; indeed, cesarean deliveries have now become the most common of all major surgical procedures currently being undertaken under the country's National Health Insurance (NHI) program. Although considerable controversy remains as to whether elective CS can provide any benefits, either to the newborn child or to the parturient [2,3], the medical costs associated with the high CS rate in Taiwan have nevertheless become a tremendous financial burden on the island's finite healthcare resources.

In order to ensure the optimum level of quality care for pregnant women and their infants, the Bureau of NHI (BNHI) has placed the CS rates of all providers under close scrutiny, adopting actual quarterly average rates by region and/or hospital accreditation levels, as a means of evaluating hospital/physician obstetric practice patterns [4]. However, numerous studies have consistently provided evidence to show that differences in case mixes among patient populations of different healthcare providers in a wide range of settings may lead to bias in the judgment of whether a provider truly has a high CS rate. Those studies suggest that prior to any comparison being undertaken between different providers, all CS rates should be adjusted for risks [5-9]. A pioneering study in Taiwan by Hsu et al. [10] pointed out the unfairness of using crude CS rates as a means of comparing hospital performance within the Taipei Municipal Hospital System, and also the inappropriateness of drawing conclusions based on crude rates on the link between hospital practice patterns and quality of care.

Prior research into risk-adjusted CS rates tended to focus on profiles of hospital CS rates; thus, to the best of our knowledge, no study has yet been carried out on case mix adjustment based upon profiles of individual physicians' CS rates. This clearly hinders the efforts by policymakers to develop effective interventions aimed at lowering the CS rates at the physician level. This study therefore took advantage of a unique dataset which merges birth certificate data with the NHI claims data, to determine the impact that adjustment for patient-specific risk factors has on CS rates among different physicians in Taiwan. The findings of this study will not only facilitate more-accurate comparisons of CS rates among physicians, but can also help policymakers target their interventions at specific physicians.

## Methods

### Data collection

All live births (*n *= 176,399) occurring in either hospitals or obstetrics/gynecology clinics between 1 January and 31 December 2003, and for whom birth certificate data could be linked with the NHI claims data, were selected as the sample for this study. The mother's date of birth, along with her unique personal identification number, provided the link between the birth certificate data and the NHI claims data.

The birth certificate dataset contains various parental demographics (including age, the highest education level achieved, marital status, and county of residence), infant gestational age (in weeks), birth weight (in grams) and gender, as well as details on multiple pregnancies and the mother's gravidity. The NHI claims data contain information on all deliveries occurring in NHI-contracted hospitals and clinics (over 92% of all healthcare institutions), including the method of delivery, the characteristics of the hospital/clinic and attending physicians, as well as one principal diagnosis code, and up to four secondary diagnosis codes for each hospital admission, from the International Classification of Disease, Ninth Revision, Clinical Modification (ICD-9-CM).

Those cases with missing data (*n *= 924), and those cases for which delivery was carried out by physicians who had fewer than 20 deliveries in 2003 (*n *= 2964), were excluded from the sample. The reason for the exclusion of the latter group of physicians was to ensure that all physicians included within this study had adequate obstetrics practical experience. We were ultimately left with a study sample of 172,511 deliveries for analysis in this study, comprising 60,079 births by CS and 112,432 vaginal deliveries.

The availability of unique physician identifiers within the claims data for each medical claim submitted enabled us to identify the same physician carrying out one or more deliveries in 2003; data for that year indicated that 1031 physicians delivered the babies of the 172,511 sampled patients. We divided the physicians into four equivalent groups based upon the quartile distribution of their crude (actual) CS rates during the period of study. Those physicians whose CS rates fell into the top quartile had the highest crude CS rates, while those falling into the bottom quartile had the lowest crude CS rates.

The institutional review board (IRB) at Taipei Medical University, Taipei, Taiwan granted ethical approval for the study.

### Variable definitions

'Case mix' represents those factors demonstrated in the literature to increase the risk of CS delivery. In this study, we categorized these factors into obstetric, pregnancy, and other risk factors.

#### Obstetric risk factors

Numerous studies have documented a breech presentation, dystocia, and fetal distress as obstetric factors which are indications for a CS [5,11-14]; however, while these are common indications for a CS, dystocia and fetal distress diagnoses are often very subjective, and, indeed, are often not risk factors in themselves. The inclusion of these factors in the regression, along with preexisting risk factors, may well have masked many important differences by 'adjusting away' subjective practice differences among physicians. Furthermore, since dystocia may be related to fetal macrosomia, and fetal distress may be related to various other conditions (including diabetes, hypertension, and collagen vascular disease), this may once again have introduced potential redundancies into the regression.

There is also a lack of standard clinical criteria for defining dystocia and fetal distress [8,10], with obstetricians in different healthcare institutions possibly applying the terms to quite-different conditions; thus, the variability in the proportions of patients diagnosed with dystocia and fetal distress may be partially attributable to differences in defining these conditions, rather than differences in the physician/patient mix. We therefore selected malpresentation (ICD-9-CM codes 652, 761.7, 763.0, or 763.1), a prolapsed cord (663.0), antepartum hemorrhage, abruptio placenta, and previa placenta (641, 762.0 or 762.1) to better define and represent the obstetric risk factors likely to result in a CS.

#### Pregnancy risk factors

A number of independent factors, all of which seem clinically relevant and are known to physicians prior to delivery, were included in this study. A woman was considered to have a pregnancy risk factor if she had one or more of the following conditions: a previous CS history (654.2), genital herpes (647.6), diabetes mellitus (648.0, 648.8, or 775.0), anemia (648.2), cardiac disease (648.5 or 648.6), arterial hypertension (642.0, 642.1, 642.2, 642.3, 642.9, or 760.0), eclampsia/preeclampsia (642.4, 642.5, 642.6, or 642.7), polyhydramnios/oligohydramnios (657.0 or 658.0), infection of the amniotic cavity (658.4), a congenital/acquired abnormality of the cervix or vagina (654.6 or 654.7), iso-immunization with Rh antigen (656.1), insufficient or excessive fetal growth (656.5 or 656.6), cerebral occlusion-hemorrhage (430, 431, 432, 433, or 434), premature labor (644), a multiple gestation (651), premature rupture of the membrane (658.1), or cervical incompetence (654.5).

#### Other risk factors

Certain variables from the database were also recorded to create clinically meaningful categories. These variables included maternal age (in years) at the time of the infant's birth, infant gender, and parity. Maternal ages were grouped into < 20, 20–34 and ≥ 35 years. Since birthweight and gestational week are highly correlated, to prevent redundancy by including both parameters, gestational age was selected to capture the effect of preterm (< 37 weeks) or postdate delivery (≥ 42 weeks). Insufficient or excessive fetal growth (656.5 or 656.6) was also included as a pregnancy risk factor to further distinguish the risk of intrauterine growth retardation or macrosomia for a given gestational age. Parity was recorded as whether or not a mother was parous.

### Statistical analysis

All of the statistical analyses within this study were performed using the SAS statistical package (SAS System for Windows, version 8.2, Cary, NC). The χ^2 ^test was first performed as a means of assessing whether there were any significant variations in the distribution of patient population risks across the physician quartiles, and second, to evaluate whether there were any significant differences, again by physician quartiles, in the cesarean delivery rates among women for each of the risk factors.

A univariate analysis was first carried out as the primary method of calculating the odds of a cesarean delivery, in order to determine whether there was any association with the potential risk factors. Stepwise logistic regressions were then conducted on parturients delivered by physicians in all four quartiles (referred to as model 1) to first develop a predictive model, and second, as a means of minimizing the number of predictive risk factors within the formula. A *p *value of < 0.05 was required for entry of a risk factor into the model. Given the sufficiently large volume of sample patients adopted for the current study, it was extremely unlikely that any important variables would have been overlooked.

Having computed the predicted risk of CS for each woman based upon the predictive model, these predicted risks were then aggregated by physician to determine the expected (risk-adjusted) CS rate and 95% confidence interval (CI) for each physician. The actual rates were then compared with the expected CS rates for each physician so as to determine how many of the physicians were below, within, and above the predicted CI in each physician quartile.

The main problem with risk adjustment, however, is the way in which an appropriate 'gold standard' is applied. Deriving a logistic equation and then applying it back to the same population from which it was derived is a well-accepted technique; however, there is a certain circular logic to this method, since the gold standard is partially defined by practices that are themselves outliers, such as the case in model 1.

Since the average CS rates for physicians in quartile 4 were as high as 52%, we therefore treated physicians in quartile 4 as outliers and repeated the logistic regression using only those parturients delivered by physicians in quartiles 1, 2, and 3. The results of this analysis are referred to as model 2. Based upon the regression results from model 2, we then recalculated the predicted CS rate and the 95% CI for each physician in the four quartiles, and again determined how many of the physicians were below, within, and above the predicted CI in each physician quartile. Our intuition was that there would be even-greater variation in predicted cesarean section rates under model 2 than under model 1.

## Results

The total number of deliveries and mean CS rates are summarized in Table 1 by physician quartiles. Of all the deliveries carried out in Taiwan in 2003, the overall CS rate was 36%, with the mean CS rates being 21% for physician quartile 1; 32% for quartile 2; 39% for quartile 3; and 52% for quartile 4.

In total, 258 physicians delivered 40,973 babies in the first quartile, 257 physicians delivered 54,464 babies in the second quartile, 258 physicians delivered 45,456 babies in the third quartile, and 258 physicians delivered 31,618 babies in the fourth quartile.

Table 2 presents the CS rate and frequency for each risk factor by physician quartile. As expected, the presence of risk factors considerably increased from the lower quartile to the higher quartiles. As results of the χ^2 ^test showed, there were statistically significant variations in the distribution of patient population risks across physician quartiles in almost all of the risk factors, with the exceptions of a prolapsed cord, genital herpes, diabetes mellitus, arterial hypertension, polyhydramnios/oligohydramnios, and congenital/acquired abnormalities of the cervix or vagina.

Similarly, the χ^2 ^test in Table 2 also indicated that with the notable exception of those cases involving a prolapsed cord, on presentation of each of the risk factors, the decision to perform a surgery significantly increased from the lower quartile to the higher quartiles.

Results of the univariate analysis in Table 3 suggest that those with a significantly greater likelihood of undergoing a CS included parturients who were multiparous, had a multiple gestation, were preterm, were aged over 35 years, were undergoing premature labor, or had malpresentation, a prolapsed cord, antepartum hemorrhage, a previous history of CS, insufficient or excessive fetal growth, genital herpes, diabetes mellitus, had anemia, cardiac disease, arterial hypertension, eclampsia/preeclampsia, polyhydramnios/oligohydramnios, an infection of the amniotic cavity, a congenital/acquired abnormality of the cervix or vagina, or cervical incompetence.

All of the risk factors included within the univariate analysis were retained in the stepwise regression analysis for adjusting the CS rates. The results for model 1 in Table 3 indicate that after controlling for all the risk factors within the model, the adjusted odds ratio for most of the obstetric- and pregnancy-related complications were of considerably greater magnitude compared to the unadjusted risks, while those of premature labor and maternal age were reduced. However, with the exceptions of premature rupture of the membrane and parity, the direction of the relationship between the CS rate and all other variables remained unchanged. Nulliparous women and women with premature rupture of the membrane were no longer protective against CS, but instead, incurred increased risk.

The results of the stepwise logistic regression, using parturients delivered by physicians in quartiles 1, 2, and 3, are presented in model 2 of Table 3. Within the model, with the exception of cervical incompetence, the direction of the relationship between the CS rate and all other risk factors remained unchanged, with the adjusted odds ratio for most of the obstetric- and pregnancy-related complications being of similar magnitudes when compared to the results of model 1. Cervical incompetence was excluded from the prediction of the CS rate in model 2.

Both the likelihood ratio and the Hosmer-Lemeshow test were significant in models 1 and 2, at *p *< 0.0001, indicating that while the risk factors considered in both models were significant, in terms of predicting the probability of a CS, the fit was nevertheless imperfect. However, the number of concordant pairs was very high, with the respective *c*-statistic for models 1 and 2 respectively being 0.870 and 0.872, thereby indicating that the predictive accuracy of the logistic model was good in both models.

Each of the models derived from the stepwise logistic regression analysis in Table 3 was used to generate a predicted CS rate for each physician based upon the patient mix for that physician. The actual CS rates, the predicted rates generated by models 1 and 2, and the percentages of physicians whose actual rates were below, within, or above the predicted 95% CI are presented in Table 4 by physician quartile.

The overall results from model 1 show that of 1031 physicians, 448 physicians (43.45%) had CS rates which were above the predicted CI; as expected, the proportion of physicians whose CS rates were above the predicted 95% CI increased for the higher quartiles. In the lowest quartile, no physician's actual rate was above the predicted CI, while in the highest quartile, the actual rates of as many as 218 physicians (84.85%) were above the predicted CI.

Of the 448 physicians whose actual rates were greater than the predicted CI, 230 physicians (51.34%) were not in the highest quartile of actual rates; on the other hand, of the 446 physicians whose actual rates were below the predicted CI, 212 physicians (47.53%) were not in the lowest quartile.

When a stricter standard was applied to the assessment of physician performance, the results from model 2 in Table 4 indicated that compared to the results of model 1, there were increases in the numbers of physicians whose CS rates were above the predicted CI in each quartile. Overall, as many as 60% of the physicians were found to have higher CS rates than the predicted CI.

In addition, within model 2, CS rates of 24 physicians in the lowest quartile were now higher than the predicted CI. Moreover, the actual rates of all the physicians in the highest quartile were above the predicted CI, and indeed, the CS rates of no physicians in either quartile 3 or quartile 4 were below the predicted CI.

## Discussion

CS rates have been used for a decade or so as a means of comparing obstetric practices across different providers, with many studies throughout this period having documented the need for risk adjustment for fairer comparisons among providers [5-10]. In 2000, the American College of Obstetricians and Gynecologists (ACOG) recommended that all CS rates should be risk-adjusted (case-mix adjusted) prior to any comparisons being made [15]; however, while this may be desirable, in many areas or countries, such adjustment is usually not feasible, largely due to the lack of adequate data sources.

The CS rate in Taiwan has hovered between 32% and 34% since implementation of the NHI program in 1995. Although lower than the respective CS rates of 36% and 40% found in Brazil and Chile, the CS rate in Taiwan is nevertheless much higher than the 15% limit recommended by the World Health Organization [16].

Inappropriate CS procedures increase maternal and neonatal morbidity and healthcare costs; however, the BNHI has continually adopted crude CS rates as its measure of quality in obstetrics management, and this can clearly lead to unfair assessments of those providers who are serving high-risk populations, since these providers will obviously have higher CS rates, and will therefore appear to be providing a lower quality of care.

By examining all 172,511 births in Taiwan in 2003, the present study took advantage of a unique population-based dataset which merges birth certificate data with the NHI claims data of this population to show how adjustments of patient risk factors can significantly change the overall assessment of a physician's obstetrics performance.

Our data specifically demonstrate that more than half of all physicians with inappropriately high rates were not in the highest quartile, and that these would be overlooked if the assessment was only targeted at those physicians in the highest quartile of unadjusted rates. Conversely, we also found that some physicians (40 of 258 physicians) in the highest quartile were actually providing obstetric care that was appropriate to the risk. However, our data also demonstrate that when stricter standards were applied to the evaluation of physician performance, a discernibly higher proportion of physicians were seen to be producing inappropriately high CS rates. In particular, we suggest that all of the physicians in quartile 4 should be placed under close scrutiny.

The CS prediction model adopted for this study was the logistic regression method, which is very similar to that used in analyses undertaken in the US and Europe [5-9].

In general terms, the factors associated with CS are largely similar between various regions or countries; however, due to differences in the data sources or specific patient populations under examination, the odds ratios associated with each of the variables may vary.

In contrast to many prior studies [6-8], our results show that prior to adjustment, multiparous women are at higher risk of cesarean delivery, mainly attributable to their prior history of a CS. Our data show that of the 87,777 births to parous women, 31,747 (36.17%) were delivered by CS, and 17,594 (55.42%) of these were repeat CSs. The data also reveal that of the 84,734 nulliparous women, 28,332 (33.44%) underwent a CS.

There are limitations to this study which should be noted. First, the possibility may exist for differential misclassification in obstetric/pregnancy risks for CS and vaginal delivery, since women who deliver vaginally tend to underreport complications much more than do those who undergo a CS. Second, the analysis in this study relied upon linked administrative data; however, some of the prior studies evaluating the use of a CS indicated that the use of administrative data may yield similar or higher discrimination than models based upon medical records [17,18]. It has been noted that across different medical institutions, medical record coders may have inconsistent coding practices, while clinicians may have very different views on diagnosis. Third, we do not have access to information on whether an obstetrician collaborated with midwives or family physicians who themselves do not have training/privileges to perform a CS. Such collaboration may have inflated an obstetrician' s apparent CS rate and elevated him/her into the uppermost percentile. Finally, our study was based on a dataset of linked birth certificate and NHI claims data for a single country, which may not be representative of other regions or countries. Thus, we recommend caution when interpreting these results, or attempting to apply them to other regions or countries.

## Conclusion

Our study arrived at the same general conclusions reported in the literature in both the US and Europe: that is, comparisons of unadjusted CS rates might not validly reflect the quality of obstetric care being provided, and may ultimately lead to biased judgments by health care purchasers. It is worth noting that the same issues which apply to obstetrics care in the US and Europe also apply to non-Western cultures, such as Taiwan.

While it is clearly important to be able to identify the risk factors associated with CS, in order to optimize the utilization of both resources and personnel, risk adjustment using average performance as a benchmark is not necessarily a true measure of quality [19]. Our study results also show that when we changed the standard of quality assessment, the evaluation results changed.

This study represents the first attempt within a developing country to use a population-based dataset as a means of calculating risk-adjusted CS rates in order to identify those physicians who are above or below the average practice in a high CS rate setting, which prevails in Taiwan. We believe that this represents a cornerstone with regard to the design of effective strategies for the monitoring of the 'epidemic' CS rates currently existing in Taiwan.

## Abbreviations

BNHI, Bureau of National Health Insurance; CI, confidence interval; CS, cesarean section; NHI, National Health Insurance.

## Competing interests

The authors declare no financial or non-financial associations exists that might pose a conflict of interest in this study.

## Authors' contributions

The individual contributions of each of the authors are as follows. C.-H. Tang identified the study issue, designed the methods, analyzed the data, and drafted the manuscript. C.-S. Hsu, H.-W. Su, and M.-J. Chen helped conceptualize the ideas and synthesize the analyses. H.-I. Wang performed the statistical analysis and participated in the writing of the manuscript. H.-C. Lin assisted with the analyses and editing. All of the authors helped to interpret the findings and revise the manuscript drafts, and approved the final manuscript.

## Pre-publication history

The pre-publication history for this paper can be accessed here:



## Figures and Tables

**Table 1:** Mean cesarean section (CS) rates by physician quartile

Variables	Physician quartile				
	
	Quartile 1	Quartile 2	Quartile 3	Quartile 4	Total
No. of physicians	258	257	258	258	1031
No. of deliveries	40,973	54,464	45,456	31,618	172,511
Mean CS rate	0.21	0.32	0.39	0.52	0.36
Standard deviation	0.06	0.02	0.02	0.08	0.13
Maximum	0.28	0.35	0.43	0.89	0.89
Minimum	0.00	0.29	0.35	0.44	0.00

**Table 2:** Distribution of selected parturient risk factors by physician quartile

	Physician quartile												χ^2 ^test on distribution of patient risks	χ^2 ^test on CS rate for patient risks
			
	Quartile 1			Quartile 2			Quartile 3			Quartile 4				
	
	No.	%	CS rate	No.	%	CS rate	No.	%	CS rate	No.	%	CS rate	*p *value	*p *value
Obstetric factors														
Malpresentation	2314	5.65	97.84	3881	7.13	99.15	3559	7.83	98.65	2982	9.43	99.56	< 0.0001	< 0.0001
Prolapsed cord	12	0.03	91.67	31	0.06	100.00	15	0.03	86.67	13	0.04	84.62	0.14	0.20
Antepartum hemorrhage, abruptio/previa placenta	335	0.82	89.55	811	1.49	94.45	783	1.72	93.49	982	3.11	97.76	< 0.0001	< 0.0001

Pregnancy factors														
Previous CS history	3373	8.23	95.67	5879	10.79	96.19	6233	13.71	97.83	5201	16.45	98.96	< 0.0001	< 0.0001
Insufficient or excessive fetal growth	232	0.57	64.66	379	0.70	73.09	365	0.80	84.93	280	0.89	88.21	< 0.0001	< 0.0001
Genital herpes	12	0.03	50.00	21	0.04	80.95	23	0.05	56.52	21	0.07	95.24	0.10	0.0066
Diabetes mellitus	185	0.45	35.14	194	0.36	56.19	175	0.38	64.00	117	0.37	79.49	0.11	< 0.0001
Anemia	583	1.42	42.88	745	1.37	58.66	486	1.07	63.58	472	1.49	81.14	< 0.0001	< 0.0001
Cardiac disease	13	0.03	46.15	42	0.08	64.29	30	0.07	50.00	31	0.10	80.65	0.0047	0.0477
Arterial hypertension	175	0.43	41.71	217	0.40	63.59	162	0.36	61.73	111	0.35	78.38	0.25	< 0.0001
Eclampsia/preeclampsia	282	0.69	62.41	369	0.68	82.66	403	0.89	83.62	330	1.04	92.73	< 0.0001	< 0.0001
Polyhydramnios/oligohydramnios	92	0.22	33.70	107	0.20	60.75	106	0.23	65.09	74	0.23	67.57	0.56	< 0.0001
Infection of the amniotic cavity	57	0.14	49.12	82	0.15	74.39	76	0.17	92.11	85	0.27	87.06	0.0001	< 0.0001
Congenital/acquired abnormality of cervix/vagina	7	0.02	42.86	14	0.03	79.57	16	0.04	93.75	13	0.04	100.00	0.22	0.0053
Premature labor	1269	3.10	24.11	1798	3.30	42.32	1626	3.58	53.75	1186	3.75	63.83	< 0.0001	< 0.0001
Premature rupture of membranes	1283	3.13	19.56	1974	3.62	26.55	1715	3.77	31.66	1092	3.45	48.08	< 0.0001	< 0.0001
Cervical incompetence	8	0.02	12.50	22	0.04	27.27	23	0.05	39.13	40	0.13	75.00	< 0.0001	0.0002

Other risk factors														
Nulliparous	19,726	48.14	20.86	26,622	48.88	29.38	22,882	50.34	37.24	15,504	49.03	50.79	< 0.0001	< 0.0001

Gestational weeks														
< 37	3008	7.34	34.71	4419	8.11	43.77	3932	8.65	54.43	2983	9.43	64.30	-	< 0.0001
≥ 42	131	0.32	22.90	198	0.36	36.36	145	0.32	40.69	102	0.32	50.00	< 0.0001	0.0002

Multiple gestation	975	2.40	65.33	1400	2.57	77.57	1432	3.15	85.61	1441	4.56	90.35	< 0.0001	< 0.0001
Maternal age ≥ 35 yr	4212	10.28	34.59	5752	10.56	47.79	5699	12.54	54.17	4753	15.03	64.09	< 0.0001	< 0.0001

**Table 3:** Final model of the stepwise regression analysis

Risk factor		Model 1*		Model 2*
			
		Crude OR (95% CI)	Adj. OR (95% CI)	Adj. OR (95% CI)
Multiple gestation		8.5 (7.9–9.1)	12.4 (11.5–13.5)	11.7 (10.7–12.8)
Multiparous		1.1 (1.1–1.2)	0.4 (0.4–0.4)	0.4 (0.4–0.4)
Gestation period (weeks)	< 37	1.9 (1.8–2.0)	-	-
	≥ 42	1.1 (0.9–1.3)	2.2 (1.8–2.7)	2.4 (1.9–2.9)
Maternal age (years)	< 20	0.4 (0.4–0.5)	0.6 (0.5–0.6)	0.6 (0.5–0.6)
	≥ 35	2.2 (2.1–2.2)	1.9 (1.9–2.0)	1.9 (1.8–2.0)
Malpresentation		206.8 (175.4–243.8)	468.0 (396.6–552.3)	481.2 (404.3–572.8)
Prolapsed cord		24.5 (9.9–60.7)	61.7 (24.4–156.1)	107.0 (32.9–347.9)
Antepartum hemorrhage, abruptio placenta, and previa placenta		35.3 (30.0–41.6)	95.6 (81.0–112.8)	86.9 (72.5–104.2)
Previous CS history		100.8 (92.6–109.7)	291.0 (266.6–317.7)	287.2 (261.6–315.3)
Insufficient or excessive fetal growth		6.9 (6.0–7.9)	13.1 (11.4–15.2)	13.0 (11.0–15.2)
Genital herpes		5.0 (3.0–8.2)	10.4 (6.1–17.7)	8.0 (4.4–14.5)
Diabetes mellitus		2.4 (2.1–2.8)	3.1 (2.5–3.7)	2.8 (2.3–3.5)
Anemia		2.9 (2.7–3.1)	3.6 (3.2–4.0)	3.3 (2.9–3.7)
Cardiac disease		3.2 (2.2–4.6)	6.0 (4.0–9.0)	5.1 (3.2–8.3)
Arterial hypertension		2.8 (2.4–3.3)	4.4 (3.7–5.3)	4.4 (3.6–5.4)
Eclampsia/preeclampsia		8.2 (7.2–9.4)	15.4 (13.4–17.8)	14.6 (12.5–17.1)
Polyhydramnios/oligohydramnios		2.5 (2.1–3.1)	3.5 (2.7–4.4)	3.7 (2.8–4.8)
Infection of amniotic cavity		6.5 (5.0–8.6)	11.3 (8.5–15.1)	11.5 (8.3–16.0)
Congenital/acquired abnormality of cervix or vagina		9.8 (4.6–20.8)	19.8 (8.9–44.0)	19.6 (8.7–44.6)
Premature labor		1.6 (1.5–1.7)	1.5 (1.4–1.6)	1.6 (1.4–1.7)
Premature rupture of the membrane		0.8 (0.8–0.9)	1.4 (1.3–1.5)	1.4 (1.3–1.5)
Cervical incompetence		1.8 (1.2–2.8)	2.8 (1.7–4.6)	-

Note: * OR, odds ratio; CI, confidence interval.

**Table 4:** Predicted cesarean section rates by physician quartile

	Physician CS rates		Proportion below predicted CI		Proportion within predicted CI		Proportion above predicted CI	
	
Quartile	Mean predicted rate	95% CI	No.	%	No.	%	No.	%
Model 1								
Quartile 1	0.28	0.27–0.28	234	90.70	24	9.30	-	-
Quartile 2	0.33	0.33–0.34	177	68.87	42	16.34	38	14.79
Quartile 3	0.37	0.36–0.37	35	13.57	31	12.02	192	74.41
Quartile 4	0.44	0.43–0.45	-	-	40	15.50	218	84.50

Total	0.35	0.35–0.36	446	43.26	137	13.29	448	43.45

Model 2								
Quartile 1	0.26	0.25–0.27	183	70.93	51	19.77	24	9.30
Quartile 2	0.31	0.31–0.32	99	38.52	41	15.96	117	45.52
Quartile 3	0.35	0.34–0.36	-	-	39	15.12	219	84.88
Quartile 4	0.42	0.41–0.43	-	-	-	-	258	100

Total	0.33	0.33–0.34	282	27.35	131	12.71	618	59.94
